# Does Fungal Endophyte Infection Improve Tall Fescue’s Growth Response to Fire and Water Limitation?

**DOI:** 10.1371/journal.pone.0086904

**Published:** 2014-01-31

**Authors:** Sarah L. Hall, Rebecca L. McCulley, Robert J. Barney, Timothy D. Phillips

**Affiliations:** 1 Department of Plant and Soil Sciences, University of Kentucky, Lexington, Kentucky, United States of America; 2 Community Research Service, Kentucky State University, Frankfort, Kentucky, United States of America; Centro de Investigación y de Estudios Avanzados, Mexico

## Abstract

Invasive species may owe some of their success in competing and co-existing with native species to microbial symbioses they are capable of forming. Tall fescue is a cool-season, non-native, invasive grass capable of co-existing with native warm-season grasses in North American grasslands that frequently experience fire, drought, and cold winters, conditions to which the native species should be better-adapted than tall fescue. We hypothesized that tall fescue’s ability to form a symbiosis with *Neotyphodium coenophialum*, an aboveground fungal endophyte, may enhance its environmental stress tolerance and persistence in these environments. We used a greenhouse experiment to examine the effects of endophyte infection (E+ vs. E−), prescribed fire (1 burn vs. 2 burn vs. unburned control), and watering regime (dry vs. wet) on tall fescue growth. We assessed treatment effects for growth rates and the following response variables: total tiller length, number of tillers recruited during the experiment, number of reproductive tillers, tiller biomass, root biomass, and total biomass. Water regime significantly affected all response variables, with less growth and lower growth rates observed under the dry water regime compared to the wet. The burn treatments significantly affected total tiller length, number of reproductive tillers, total tiller biomass, and total biomass, but treatment differences were not consistent across parameters. Overall, fire seemed to enhance growth. Endophyte status significantly affected total tiller length and tiller biomass, but the effect was opposite what we predicted (E−>E+). The results from our experiment indicated that tall fescue was relatively tolerant of fire, even when combined with dry conditions, and that the fungal endophyte symbiosis was not important in governing this ecological ability. The persistence of tall fescue in native grassland ecosystems may be linked to other endophyte-conferred abilities not measured here (e.g., herbivory release) or may not be related to this plant-microbial symbiosis.

## Introduction

Plant species may be considered invasive when they successfully spread outside their native range [1], and may use a number of mechanisms to gain competitive advantage over native species. They may be released from their natural enemies and thus able to thrive better in the new environment [2], they may simply be better competitors for resources in disturbed environments [3], and/or they may use plant-soil feedbacks, including so-called “novel weapons” [4], to negatively impact co-occurring native plants. In some cases, these effects may not be coming from the plants alone, but may be mediated by their association with microorganisms [5]. Many plant functional traits have been linked to association with bacterial and fungal microorganisms (reviewed in [6]), with fungal endophytes of grasses (in the family Clavicipitaceae) being one of the most studied associations [7], [8], [9]. Association with these fungal endophytes has been linked to success of the invasive annual Italian ryegrass [10], including conferring increased herbicide resistance [11] (but see [12]). In addition, many of the grass functional traits affected by fungal endophytes could be considered traits that make the grasses more competitive [7] and potentially more able to successfully persist and/or invade novel habitats.

Tall fescue (*Schedonorus phoenix* (Scop.) Holub) is a non-native C_3_ grass species, introduced in the late 1800’s which now covers 14 million hectares in the United States, with its adapted range being the entire eastern U.S. and areas within the Pacific Northwest [13], [14]. In areas being managed for native warm-season grasslands in North America, tall fescue is considered an undesirable species, in part because it can outcompete native grassland species [15], and in part due to negative effects on wildlife [16]. Prescribed fires are used widely in management of many grasslands today [17] [18], either alone or in combination with herbicide application [19]. The persistence of tall fescue in what are largely C_4_-dominated grass systems, which often undergo frequent fire and/or water limitation (e.g. [20]), suggests it is tolerant of these conditions. Other non-native cool-season perennial grasses have been successfully eliminated with prescribed burns [21], but this has not been the case for tall fescue, which experienced no growth suppression following prescribed burns in the field [16], [19], [22]. One factor that may impact growth response of tall fescue to management practices such as prescribed fire is its frequent association with the fungal endophyte *Neotyphodium coenophialum* (whose presence was unknown in the studies reported in [16], [19], [22]).

The tall fescue-*Neotyphodium* symbiosis is known to increase tall fescue’s stress tolerance over that of endophyte-free (E−) individuals [8], [23]. Endophyte presence within tall fescue populations can vary across the landscape: within a single field, some areas may have no individuals infected, whereas in other areas, all individuals present are infected. Extensive surveys of tall fescue populations in North America show that on average >50% of tall fescue tillers in an area test positive for endophyte presence [24], [25], [26]. Surveys of 17 tall fescue pastures being targeted for restoration across the state of Kentucky found all but one had endophyte infection frequencies (EIF) >80% [27]. ‘Kentucky-31’, the variety of tall fescue that is most common in pastures in this region, has a higher occurrence of fungal endophyte symbiosis than other varieties [25]. The physiological benefits to tall fescue of hosting *N. coenophialum* are thought to be most pronounced under water [28], [29], [30] or nutrient deficiency [31] (but see [32]), and the fungus may actually serve as a physiological drain or sink when the plant is not under such stress [31]. Endophyte-infected (E+) fescue has been shown to have larger belowground biomass compared to E− tall fescue [33], [34], [35], which could serve as a greater resource from which to recover following management activities that negatively impact aboveground growth of the plant. E+ plants have also been shown to respond to increased nutrient availability (which may be influenced by management) more than E− plants [28], [32].

Prescribed fire is used as a management tool in many grasslands, and can affect the abiotic and biotic components of the ecosystem. In mesic grasslands effects of fire on the abiotic environment include increased light levels and decreased soil moisture at the surface, and increased nutrient availability [36]. These abiotic effects may in turn affect biotic components of the grassland systems where they occur. Burning has been shown to reduce cover of some non-native species [37], [38], [39] and C_3_ grasses [38], [39], [40] while simultaneously increasing native warm-season grass tillering [41]. The behavior of fire (which determines impacts to the abiotic environment and effectiveness as a management tool) can also be impacted by the vegetation present [42], and areas dominated by C_3_’s, like tall fescue, often experience reduced intensity of early spring burns [43] [18], as they may have already begun to grow. In some ways, the effects of prescribed fire on tall fescue might be similar to those of grazing (e.g. removal of aboveground biomass), but comparative effects of these two common grassland disturbances most likely vary depending on the severity or intensity of the events and their distribution in space and time [44]. Prior studies have shown that fungal endophyte presence within tall fescue can alter herbivory [45] and improve plant persistence and performance under grazed conditions (e.g. [46], [47], [48], [49]). However, we are aware of no studies examining prescribed fire and its interaction with *N. coenophialum* on tall fescue survival and regrowth.

Given that fire has been shown to affect some of the same abiotic parameters also known to be important in determining whether endophyte symbiosis increases tall fescue’s competitive ability or reduces it (e.g., increases light availability, lowers soil moisture, increases nutrient availability), we wanted to explore whether endophyte infection confers greater tolerance to fire. Given previous research indicating physiological benefits of *Neotyphodium* being most pronounced under water stress, we also incorporated two levels of water availability in the experiment.

We designed a controlled greenhouse experiment to test differences in growth following prescribed burn and water availability treatments for E+ and E− tall fescue. This experiment used established tall fescue plants (variety Kentucky-31, either with (E+) or without (E−) the common toxic strain of *N. coenophialum*) to which we applied a water availability treatment, providing half the plants with adequate water supply (‘wet’), and half the plants with half as much water (‘dry’). We included an unburned control, a single burn treatment (1x), and a two burn treatment (2x). Based on prior work that suggests the fungal endophyte symbiosis is generally mutualistic, especially under stressful abiotic conditions, we hypothesized E+ plants would have higher biomass and growth compared to E− plants, and that differences would be most pronounced under the dry treatment. We also thought differences between the E+ and E− plant responses would be greatest for those individuals that received the presumably more stressful 2x burn treatment.

## Materials and Methods

This experiment consisted of a full factorial design, with E− and E+ tall fescue, three burn treatments (1x, 2x, and unburned control), a wet and dry treatment, and six replicates per treatment combination (2×3×2×6 = 72 plants). Replicates were arranged into six randomized blocks.

### Field Collection of Plant Material

On 6 and 9 March 2009, tall fescue plants (cultivar Kentucky-31) were removed from 0.08-ha plots established in 2001 at the University of Kentucky Research Farm, Lexington, KY, USA, for the purpose of E+ and E− tall fescue seed production (n = 2 plots of each endophyte status; common toxic strain of *N. coenophialum*) (39.219167°N −86.541389°W). Prior work has shown that plant genotype can affect the nature of the grass-fungal endophyte symbiosis [50], [51], [52], [53]; however, it was beyond the scope of this study to evaluate plant genotype interactions. Individual plants that had two to three overwintering tillers were selected from E− and E+ plots. When measured in 2007, these plots were dominated by tall fescue (visual cover estimates of 92% in E+ and 84% in E−) and when tested using an immunoblot assay specific for *N. coenophialum* (Agrinostics Ltd., Watkinsville, GA) 96% of tillers tested positive for endophyte infection in E+ plots and 4% for E− [54]. PVC pipe sections (7.5cm in diameter, 22 cm in length) were placed around each individual plant, and a hammer was used to pound the pipe into the ground, leaving a 2-cm deep rim above the soil surface. Each pipe section was extracted, and contained the top 20 cm of soil and the individual tall fescue plant. These pipe sections served as pots in the greenhouse, and will be referred to as such. Each was marked as E− or E+, and plants were transferred to a greenhouse at the Kentucky State University Research Farm (38.116065°N −84.890506°W).

### Greenhouse Conditions

Plants were given ambient light with a 21°C day (12 hr) and 15.6°C night (12 hr). Pots were individually numbered, and were randomly assigned burn treatment, watering regime, and replicate number. During this initial 2.5 week growing period (from 6 or 9 March to 25 March), all pots were watered twice a week to field capacity. Pots were arranged in six randomized blocks across a single greenhouse bench. On 18 March, the number of tillers for each individual pot was counted (mean±SE = 6.9+0.3). All tillers were clipped to 4-cm height, and clipped material was kept from each pot and placed in plastic bags. The following day a wet weight was measured for all clipped material. Eight E− and eight E+ pots were randomly selected for harvesting at this time (18 March) to estimate belowground biomass prior to the experiment, and to obtain pre-treatment soil moisture levels. At this time, it had been two days since the last watering event.

### Burn Treatments, Watering Regime, and Fertilization

Senesced plant litter of native warm-season grasses (primarily switchgrass (*Panicum virgatum* L.) and little bluestem (*Schizachyrium scoparium* (Michx.) Nash)) was collected from a field at the KSU Research Farm to serve as fuel for the prescribed burn treatments, in an effort to mimic litter conditions in native grassland where tall fescue is present. It was placed in the greenhouse for one week to dry, and was then cut into approximately 3-cm pieces. In order to evaluate heat levels of the prescribed fire treatment, we made aluminum tags that were painted with different heat-sensitive paints (Tempil Inc., S. Plainfield, NJ) that change appearance at 79, 163, 246, 316, 399, and 510°C. Each tag was wrapped in aluminum foil (which melts at 644°C).

On 25 March, the first prescribed burn treatment was applied to all pots assigned to one of the two burn treatments (1x or 2x). A single heat-sensitive paint tag was anchored at the crown level of each pot with a paper clip. Pots were burned in (random) groups of 12 on a concrete floor inside the headhouse. Doors were opened on either side of the headhouse to allow for some air movement. Pots were placed such that paint tags were all in the same direction (with air flow). A 250-ml cup was used to scoop dried native grass litter (approximately 5.3 g) onto each pot. Care was taken to get as much litter as possible over each pot. A standard lighter was used to light the native grass litter in all pots for each group. Once the fire had burned out (≤1minute), paint tags were removed and marked with their respective pot number. Relative humidity inside the headhouse was 100% (it was raining outside at the time of the burns). Subsamples of native grass litter (n = 8; 250 ml each) analyzed for fuel moisture and variance in fuel amounts indicated that litter additions weighed 5.34±0.22 g (mean±standard error of the mean) and contained 9.87±0.03% moisture.

Water regime treatments began the day of the first burn, with only the wet pots receiving water immediately following the burn. On 1 April, both the wet and dry treatments were watered, the wet pots receiving 116 ml (volume of water based on long-term average of weekly March-June precipitation for Lexington, KY, www.weather.com calculated and applied based on the area of each pot) and the dry pots received half this amount, 58 ml. Pots were watered with these amounts 2–3 times per week as needed for the rest of the experiment. On 8 May, all pots were fertilized with 58 ml 10-20-10 NPK fertilizer (Peters 20-10-20 Greenhouse Fertilizer Peat-lite). Wet pots were given an additional 58 ml water without fertilizer to maintain this treatment. The same procedure was also used on 3 June to fertilize all pots.

On 12 May, the second prescribed burn treatment was applied to those pots assigned to that treatment. The same procedure was followed as for the first burn, except no paint tags were used. Relative humidity was approximately 55% at the time of the burn. Prior to burning, all plastic markers used to identify and track individual tillers were removed, and tillers that emerged after the burn treatment were marked anew (as a new “cohort”). Subsamples of fuel (n = 8; 250 ml each) weighed 6.08±0.33 and contained 5.05±0.07% moisture.

### Growth Measurements

All tillers were measured weekly (during the first month) and bi-weekly thereafter. Each tiller was measured by recording the distance from the base of the tiller to the longest green part of a leaf blade on that tiller, and lengths for all tillers in a given pot were combined to provide the total pot length for each measurement. New tillers that emerged during the experiment were measured as they appeared. Reproductive tillers were clipped to prevent seed from developing, and date of flowering was noted (these measurements occurred during the more frequent watering events). This clipped material was kept to be added to oven-dry aboveground material for biomass measurements.

On 26 Jun (100 days after experiment initiation), all control pots were harvested. Each tiller was cut at the soil surface and placed in a coin envelope. Tillers were stored cool, and double blotted onto nitrocellulose paper for endophyte testing (Agrinostics Ltd., Watkinsville, GA). Soil from each pot was sieved, and roots were removed by hand-picking. Individual tiller material and pot root material were dried at 55°C for 48 hours to obtain biomass. A 5-g subsample of soil from each pot was used to measure gravimetric soil water content. Burned pots were harvested 10 July (2x burned) and 13 July (1x burned) (114–117 days after experiment initiation, and 107–110 days after the first prescribed burn), with the same procedures followed as described for the control pots. Weights of all crown and root material were ash-corrected by placing a 0.5-g subsample of harvested biomass in a muffle furnace at 550°C for 4 hrs.

### Statistical Analyses

We analyzed all data considering the pot as the experimental unit (not individual tillers). Data (at the pot level) for the following response variables were analyzed using Proc GLM to test for effects of endophyte presence, watering regime, burn treatment, and all interactions: final total pot tiller length, number new tillers (difference between tiller number on 18 March, prior to treatment implementation, and at harvest), number reproductive tillers, oven-dry total tiller biomass, oven-dry root biomass, and total oven-dry biomass (tillers, crowns and roots). LS Means procedure for pairwise comparisons of means in SAS 9.2 (SAS Institute, Cary, NC) was used to test for significant differences between means. Means and standard errors were obtained using the Means and Standard Deviation procedure in JMP 9.0 (SAS Institute, Cary, NC).

In order to test whether the temperature of the first burn treatment affected growth, Proc GLM (SAS 9.2) was used to test for the effects of burn temperature and its interactions with water regime and endophyte presence, on the total tiller length, number new tillers, number reproductive tillers, and mean length per tiller (total pot tiller length divided by number tillers present) for burned pots as measured just prior to the second burn (12 May) for those pots assigned to the 1x or 2x burn treatment.

In order to observe trends in growth for tall fescue tillers over the entire experiment, total pot tiller length (±1 S.E.) was calculated at each of the nine measurement intervals (which took place one to three days apart between treatments) and plotted on a line graph over time. Means ANOVA procedure and Tukey-Kramer HSD were used in JMP 9.0 to test for significant differences between Wet/Dry, E+/E−, and 1x/2x/unburned controls at each of these nine measurement intervals. Relative growth rates (for total tiller length per pot) were also calculated for the different measurement intervals by dividing the difference of log lengths (at the beginning of the growth interval, and at the end of the interval) by the number of days between measurements to get the relative total tiller growth rate (cm/cm/day). For these calculations, lengths of tillers were assumed to be 1-cm immediately following a prescribed burn. These relative growth rates were also plotted on a line graph over time. Means ANOVA procedure and Tukey-Kramer HSD were used in JMP 9.0 to test for significant differences within measurement intervals between burn treatments.

## Results

### Endophyte Infection Frequency and Initial Root Biomass and Soil Moisture

Endophyte tests of tillers harvested at the end of the experiment revealed twelve of 70 pots that were not 0% (E−) or 100% (E+) endophyte-infected. Ten of the twelve were E+, two were E−. They included six each of the wet and dry water regime. Three were from the control treatment, two were burned once, and seven were burned twice. Of these twelve, only two pots had endophyte infection frequencies (EIF =  total number tillers testing positive/total number tillers) more than 50% off from what they were supposed to be (one wet regime, 2x burn, E+ pot with 40% EIF, and one dry regime, 2x burn, E+ pot with 28.6% EIF). These two pots were removed from the dataset to ensure statistical analyses were conducted on measurements from pots dominated by tall fescue of the correct endophyte status.

For the 16 randomly selected pots that were harvested prior to the implementation of water regime and burn treatments (18 March), soil moisture was significantly higher (*P* = 0.0385) for the E+ pots (mean±standard error of the mean; 17.44±1.57%) compared to E− pots (13.63±0.55%). E− plants were extracted from the field three days earlier than E+ plants, but all had been watered to field capacity two days prior to harvesting in the greenhouse, so it seems unlikely that differences in soil moisture were due to extraction date differences. Biomass of belowground material for these 16 pots harvested prior to implementation of the treatments revealed significantly (*P* = 0.0499) higher root biomass for E+ (0.98±0.15 g) compared to E− (0.63±0.06 g). The wet weight of the plant material clipped and removed at this time (any material >4-cm tall) for these same pots was not significantly different between E+ and E− plants (*P* = 0.4617). Therefore, endophyte-related differences prior to the initiation of the treatments were only apparent belowground (root biomass and soil moisture).

### Effects of Prescribed Burn Treatments on Growth

The heat-sensitive paint tags used during the first prescribed burn revealed that fire created temperatures ranging from <79°C (no paints melted) to >316°C but <399°C (the fourth paint melted). Of the 48 pots that were burned, five were <79°C, twenty-three were >79°C but <163°C, one was >163°C but <246°C, fifteen were >246°C but <316°C, and four were >316°C but <399°C. Because the number of replicates was low in several burn temperature categories, pots were categorized into those that had experienced fire temperature of <162°C and those that had experienced fire temperatures of 246–398°C in order to allow for LS Means comparisons (the one pot that was >163°C but <246°C was removed from the dataset). Burn temperature did not have a significant effect for any of the measured variables using this binned Proc GLM approach. Given the variability of temperatures within each of these ranges, we also ran linear regressions to see if any of the growth variables might be significantly correlated to fire temperature. Again, no significant relationships were identified. This lack of burn temperature effect suggests that the variability observed in fire temperature at the crown level did not result in differences in tall fescue growth as measured in a greenhouse for 48 days after the burn treatment; however, additional replicates would help further assess this claim. Surprisingly, burn treatment (1x, 2x, or control/no burn) did not significantly affect soil moisture averaged across wet/dry treatments, which was similar (10.4±0.5% for 1x, 10.8±0.5% for 2x, and 11.4±0.4% for control) in soils at the end of the experiment across burn treatments.

Burn treatment had significant main effects on total pot tiller length, number of reproductive tillers, tiller biomass, and total biomass as measured at the end of the entire experiment period (Table 1). The once and twice burned pots had greater total tiller length than the unburned control at the final harvest, while the opposite was true for number of reproductive tillers. The control had more reproductive tillers than either of the burn treatments (Table 2). Tiller biomass was greatest for the 1x burn treatment, intermediate for the control, and lowest for the 2x burn treatment (Table 2), but there was no effect of burning on root biomass (Table 1). Total biomass was greater for the control and the 1x burn treatment compared to the 2x burn treatment (Table 2).

**Table 1:** F-value and degree of significance for effects of burn treatment (1x, 2x, unburned control), water regime (dry, wet), and endophyte infection status (E+, E−) and their interactions on biomass measurements and tiller number at the final harvest.

	Burn Trtmt (2)		Water Regime (1)		Endophyte (1)		Trtmt*Water (2)		Water*Endo (1)		Trtmt*Endo (2)			Trtmt*Water*Endo (2)	
	*F*	*P>F*	*F*	*P>F*	*F*	*P>F*	*F*	*P>F*	*F*	*P>F*	*F*	*P>F*	*F*		*P>F*
Total Length	5.48	**	64.66	***	5.25	*	0.99	ns	0.24	ns	1.43	ns	0.65		ns
Number New Tillers	0.19	ns	31.02	***	1.67	ns	0.25	ns	0.04	ns	1.13	ns	0.82		ns
Number Reproductive Tillers	7.26	**	5.41	*	3.59	ns	2.37	ns	0.48	ns	1.3	ns	1.87		ns
Tiller Biomass	14.83	***	101.41	***	6.58	*	0.02	ns	0.07	ns	0.85	ns	0.18		ns
Root Biomass	0.41	ns	17.37	***	0.51	ns	0.58	ns	2.94	ns	0.69	ns	0.29		ns
Total Biomass	9.78	***	73.14	***	1.83	ns	0.14	ns	0.36	ns	1.84	ns	0.28		ns

Degrees of freedom are indicated in parentheses.

ns, not significant.

* P<0.05.

** P<0.01.

*** P<0.001.

**Table 2:** Mean measured growth response variables (±1 S.E.) for tall fescue plants exposed to 1 prescribed burn (1x), 2 prescribed burns (2x), or no prescribed burn (control), averaged across watering regimes and endophyte status.

	1x	2x	Control
Total Tiller Length (cm)	230.9±17.9 a	221.3±19.5 a	178.8±12.4 b
Number Reproductive Tillers	0.25±0.1 b	0.23±0.1 b	0.75±0.2 a
Tiller Biomass (g)	2.60±0.16 a	1.89±0.15 c	2.23±0.13 b
Total Biomass (g)	5.60±0.31 a	4.43±0.23 b	5.28±0.30 a

Parameters shown are those for which burn treatment had a significant main effect (see Table 1 above). Letters represent LS Means differences (α = 0.05) for the main burn treatment effect.

**Figure 1:**
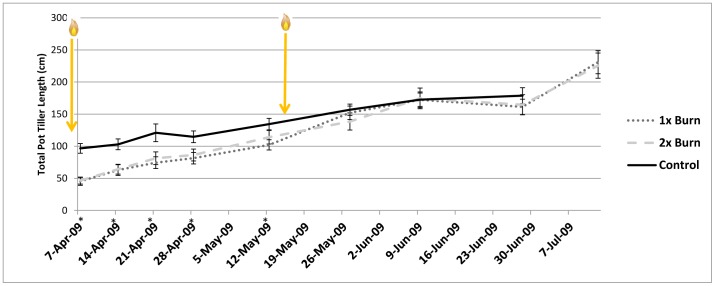
Average total pot tiller length (±1 S.E.) across the duration of the experiment, as measured at each of nine measurement intervals (measurements for all treatments were made within a two-day window for each interval). Asterisks indicate dates for which there was a significant difference between treatment means. Flame symbols indicate when the two prescribed burn treatments were applied to either both the 1x and 2x treatments for the first burn (25 March), or the 2x treatment only for the second burn (12 May).

When trends in total pot tiller length were compared over time, they varied by burn treatment for the first five measurement intervals following the 25 March prescribed burn (Figure 1). At each of the first four measurement intervals during this period, the control pots had greater tiller length compared to the burned pots, but for the last measurement during this period (12 May, just prior to the second burn), the 1x burn pots remained lower than the controls, but the 2x burn pots had become similar to the control (despite the fact that both 1x and 2x burn treatments had both received the same treatment of one burn at this point in time). By 26 May, two weeks after the second burn was performed on the 2x pots, there was no significant difference in total tiller length between any of the burn treatments. Burn treatment did not have a significant effect on total pot tiller length throughout the rest of the experiment.

**Figure 2:**
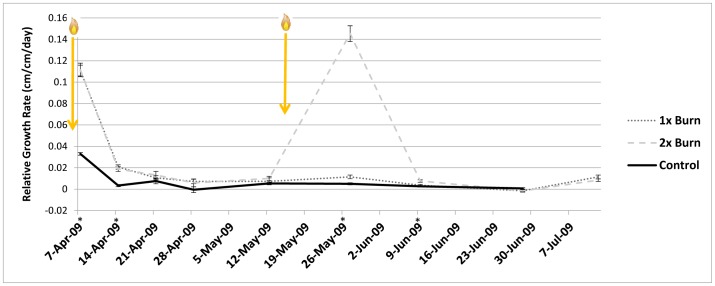
Mean relative growth rate of tillers (cm/cm/day) (±1 S.E.) across the duration of the experiment, as measured at each of nine measurement intervals (measurements for all treatments were made within a two-day window for each interval). Asterisks indicate dates for which there was a significant difference between treatment means. Flame symbols indicate when the two prescribed burn treatments were applied to either both the 1x and 2x treatments for the first burn (25 March), or the 2x treatment only for the second burn (12 May).

When relative total tiller growth rates were calculated over the experiment by burn treatment, a number of trends emerged. Immediately following the first prescribed burn, growth rates for burned pots were significantly greater compared to the controls (for the two weeks following the burn), and they remained significantly greater at the next measurement interval (third week after the burn) (Figure 2). At the fourth week after the burn these differences had disappeared, and all growth rates were similar. Similar growth rates persisted until the second burn was applied to the 2x pots. Burning a second time stimulated higher tiller growth rates in 2x pots than 1x pots for the month following the second burn (Figure 2). For the two last measurement intervals (mid to late June for all treatments, and late Jun to mid July for the 1x, 2x burned pots) there were no significant differences in growth rates.

Two pots had no aboveground live material at the final harvest- both were E− pots under the dry water regime that were burned once or twice. One of these had no aboveground material at the first measurement following the first prescribed burn, and the other had very low growth following the first burn that declined over time (no material present when second burn was applied). Given this low number of pots that experienced mortality, and the fact that one of them did have growth following the first prescribed burn, no conclusions can be made as to why these plants experienced mortality. The higher total tiller length at the final harvest for burned pots compared to the controls (Table 2) clearly suggests that burning did not negatively impact tall fescue growth, even when applied twice in a single season. Fire stimulated growth rate (but decreased biomass) initially following the first burn, with even higher growth rate following the second burn (Figure 2), and no depression in biomass accumulation (Figure 1).

### Effects of Water Regime on Growth

Water regime had the most pronounced and widespread effects on measured growth variables, being significant for all parameters (Table 1). In all cases, the dry watering regime had significantly lower measured growth responses at the final harvest than the wet treatment (Table 3). This was also true for the total tiller length at all measurement intervals, the dry pots had less tiller length than the wet (data not shown). Water regime was the only treatment that significantly affected root biomass and new tiller number, with the dry regime reducing both variables. A total of 29 reproductive tillers appeared in 24 pots over the course of the experiment, and all emerged in May. The ‘wet’ treatment had two times the number of reproductive tillers than ‘dry’ (Table 3). Water regime significantly affected date of flower during May (*P* = 0.0156), with plants under the wet treatment flowering earlier (on average, ‘wet’ plants flowered on May 6±1 days) than those under the dry treatment (average ‘dry’ date of flowering May 12±3 days). Clearly, tall fescue in those pots under the dry water regime was limited in growth compared to those under the wet water regime, as intended. Water regime had a significant effect on soil moisture of the pots at the end of the experiment, with the dry pots having significantly lower soil moisture than the wet pots (*P* = 0.0003; 9.99±0.39% (‘dry’) vs. 11.83±0.29% (‘wet’)).

**Table 3:** Mean measured growth response variables (±1 S.E.) at the final harvest for tall fescue plants exposed to Wet and Dry water regimes and averaged across burn treatments and endophyte status.

	Wet	Dry
Total Tiller Length (cm)	264.9±10.8a	155.2±10.2b
Number New Tillers	11.0±0.9a	4.0±0.8b
Number Reproductive Tillers	0.6±0.1a	0.3±0.1b
Tiller Biomass (g)	2.78±0.08a	1.72±0.10b
Root Biomass (g)	1.84±0.08a	1.36±0.08b
Total Biomass (g)	6.10±0.17a	4.16±0.18b

Parameters shown are those for which watering regime had a significant main effect (see Table 1 above). Letters represent LS Means differences (α = 0.05) for the main water regime effect.

### Effects of Endophyte Presence on Growth

Endophyte status significantly affected total tiller length (*P* = 0.0256) and final tiller biomass (*P* = 0.0129) (Table 1). In both cases, the E− tall fescue plants had greater growth than E+. Total tiller length was 193.9±12.6 cm for E+, and 225.3±14.8 cm for E−. Oven-dry tiller biomass was 2.12±0.13 g for E+ and 2.37±0.13 g for E−. Surprisingly, we did not find any significant interactions between the watering regime and endophyte presence or burn treatment (Table 1). The difference in soil moisture between E+ and E− pots observed at the initial harvest prior to implementation of the experimental treatments was no longer present at the end of the experiment (*P* = 0.3865), indicating that effects of the water regime treatment on soil moisture had over-ridden any differences present at the beginning of the experiment related to endophyte presence. The root biomass differences were also no longer significant at the end of the experiment (*P* = 0.8475).

## Discussion

Of the different treatments imposed during this experiment (endophyte status, water regime, burn), water regime had the most pronounced and consistent effect on tall fescue growth, with those plants under the dry water regime having less growth than those under the wet regime throughout the entire course of the experiment. This result was not surprising given that tall fescue is a C_3_ species that cannot perform well during warm temperatures unless adequate water is supplied [55]. However, contrary to our hypothesis and expectations, the effects of water stress imposed by the dry regime were equally detrimental for both E+ and E− plants and across burn treatments. This was surprising, given that others have observed endophyte-related differences in growth responses, especially under dry conditions [28], [29], [30], [50], [56], although in some cases these effects have been varied by host plant genotype [50], [56]. It is possible that if we had controlled for plant genotype (e.g. using genetic clone pairs of E+ and E− individuals) we would have found a different result. It is also possible that our ‘dry’ treatment was not dry enough to stimulate such endophyte effects, although it should be noted it was dry enough to depress tall fescue growth (total biomass) by approximately 32% at the end of the experiment. The relatively cool temperatures of the greenhouse and the frequency of watering (dry treatment received 50% less water than the wet treatment but was applied at the same frequency) may have played a role in not seeing the expected endophyte x water interaction.

Endophtye effects on biomass were opposite those expected (E−>E+), and as stated previously, there were no significant interactions with water regime or burn treatment. The only time E+ plants had higher biomass than E− was at the beginning of the experiment for initial root weight. E+ fescue has been shown in a number of cases to have greater shoot [28], [35], [57], [58], [59], [60] and root [33], [34], [35], [58] mass compared to E−. However, the magnitude of these differences observed in the previously mentioned studies varied widely (e.g., E+ plants 4.4% [60] to 70% [35] more biomass than E−), and there are a few studies in which no endophyte effect was observed. It is possible that enhanced root biomass reservoir might increase the ability of E+ tall fescue to regrow following aboveground biomass removal, through either fire or grazing. However, in our study, greater root biomass in E+ individuals at the start of the study appeared to have no effect on growth responses following disturbance. Similarly, endophyte presence did not affect leaf elongation, tiller density or dry weight per tiller in studies conducted by Elbersen and West [50] and Newman et al. [61]. It did result in earlier flowering in the Newman et al. study [61], but in our experiment, date of flower was not significantly affected by endophyte presence either. Some might speculate that endophyte effects are better seen in field studies than in greenhouse studies, but in a climate change experiment in the field at the same research farm where the tall fescue used here originated from (and using tall fescue propagated from seed collected in the plots from which our material came), Brosi also observed relatively few endophyte effects on tall fescue tiller growth [62]. Host plant genotype [29], [50], [56], [60], [63], [64] and fungal genotype [29], [51], [63], [64], [65] have both been shown to influence the dynamics of symbiosis within the tall fescue-*N. coenophialum* system. It may be that the combination used in our study simply does not exhibit the differences in growth seen in other cases, although it should be noted that our combination (variety ‘Kentucky-31’ and common toxic endophyte) was the same as in some of this previous work and is the most common pairing of tall fescue cultivar-endophyte on the landscape.

Physiological benefits of symbiosis with *N. coenophialum* to host plants can vary depending on soil fertility [28], [31], [32], but the results are not consistent. Cheplick et al. found higher biomass of E+ seedlings compared to E− at high nutrient levels and lower biomass for E+ at low nutrient levels [32], but Arechavaleta et al. [28] and Malinowski et al. [31] saw higher biomass for E+ at lower nutrient levels and no difference [28] or reduced biomass [31] for E+ at high nutrient levels. The plants used in the current study were grown in the relatively fertile (especially for phosphorus; see [27]) soil from which they originated. Malinowski et al. [31] and Rahman and Saiga [66] looked at tall fescue growth in response to different P levels, and our results are consistent with what both studies found in high P soils, E+ biomass was lower than E−. It may be that if we had performed this experiment in less fertile soil we would have seen a different outcome with regard to the potential endophyte effects on growth. Given the variability in growth responses in previous studies and this one, it seems there is still much to be learned about the conditions under which fungal endophyte symbiosis is strongly mutualistic for this species.

The response of tall fescue to fire might be dependent on its life history (specifically life form and bud characteristics), which Pyke et al. used to characterize plant species’ fire tolerance [42]. With tall fescue being a cryptophyte (sensu [67]), Pyke et al. predicted the growth response following fire to be neutral or positive if buds are insulated by soil, but negative if buds are closer to the surface and fire temperatures are hot enough [42]. In a review of fire effects on invasive weeds, DiTomaso et al. list cool-season perennial grasses as a category that can be controlled with burning, and while they do not specifically address tall fescue; they do cite successful reductions in Kentucky bluegrass with mid-late spring burns [21]. However, in our study, tiller length was greater for the burned pots (1x or 2x) compared to the control, but biomass (tiller and pot total) was suppressed in 2x compared to 1x or unburned control, so there was a slight reduction in material in the pots burned twice at the end of the experiment (leaf sheaths were the same lengths but apparently not as thick). The rapid growth rate following the second burn was surprising, and likely indicates that given more time prior to harvest (2x burned plants were harvested only 59 days after the second burn, but 1x burned plants were harvested 117 days after the first burn) the 2x burn pots may have regrown all, if not more than, the material lost to fire. Our study did not aim to detect whether fire could actually kill the endophyte, but when we tested for endophyte presence at the end of our experiment, we found more pots in the 2x burn treatment that differed from either 0 or 100% infection (1 such pot in control, 2 pots in 1x, and 7 pots in 2x burn). In fact, the two pots that were excluded from the study were 2x burn that had less than 100% infection. *Neotyphodium coenophialum* is known to be sensitive to heat, as heat treatments are regularly employed to remove the fungus from infected seed lots [68]. It is possible that prescribed fires may negatively affect the fungus, but more work exploring this topic is required.

Tall fescue experiences two periods of growth during a single season with a period in the mid-summer of slow growth [69], [70], and it is possible that the timing of fire might interact with the seasonal growth cycle of tall fescue to alter the plant’s response. Based on growth rates prior to burns, this experiment imposed the first burn during the period of early summer growth, and tall fescue took longer to recover compared to when the second burn applied, which occurred as the plants were entering their slower growth mid-summer period. Prescribed fires are most often conducted in February or March in the eastern U.S., which coincided with the timing of our first prescribed burn (during the initial spring growth period). Based on our data, a burn applied at this time appears to allow plenty of time for plants to recover aboveground material, and they can do so in a relatively short period of time (∼3 weeks in this greenhouse experiment). A burn during the mid-summer period (which is when the second burn in this experiment occurred) resulted in rapid recovery in length (2x burn plants had the same tiller length as 1x and unburned control within 2 weeks following the second fire), although it should be noted that the greenhouse was maintained at a daytime temperature lower than ambient summer temperatures which normally produce a “summer slump” or drop in production for tall fescue and other cool-season grasses [69], [70]. A summer prescribed burn applied to a field dominated by another C_3_ grass, Texas wintergrass, resulted in 2x higher yield of that species compared to a winter (Feb/Mar) burn or no burn [40]. A burn during the autumn growing period would allow less time for recovery before the winter dormant period, and might be predicted to reduce tall fescue dominance better over the long-term than summer or spring burns, but Madison et al. found that fall burning did not reduce tall fescue cover [16]. Our results indicate that tall fescue is able to readily recover following fire, even if applied twice in a single growing season, under wet or dry conditions, and irrespective of endophyte status.

## Conclusions

Our data suggest that regardless of endophyte status, tall fescue growth was stimulated after being burned. Water stress negatively affected tall fescue growth, and did so equally for E+ and E− plants in this experiment. When we did observe significant effects of endophyte on growth of fescue plants, it was opposite that expected, with E− plants having greater tiller length and biomass compared to E+ (one notable exception being belowground biomass prior to the treatments being applied). These results add to the growing body of literature that shows differences in E+ and E− tall fescue plant response to stress may depend on a number of factors (i.e., soil fertility, tall fescue and fungal endophyte genotype interactions, climatic factors, etc.) and are not universal across its range in the Eastern U.S. Our study indicated no apparent role of symbiosis with *Neotyphodium* in the ability of tall fescue to regrow following fire even under dry conditions such as are commonly experienced in North American grasslands. This result suggests that the persistence of tall fescue in native grassland ecosystems may be linked to other endophyte-conferred abilities not measured here (e.g., herbivory release) or not related to this plant-microbial symbiosis.
